# Prevention and Control of Small-Pox

**Published:** 1882-03

**Authors:** Eugene Foster

**Affiliations:** President of the Board of Health of Augusta, Ga.


ATLANTA

MEDICAL REGISTER.

VOL. I.] MARCH, 1882. [No. 6

### Original.

### PREVENTION AND CONTROL OF SMALL-POX.

BY EUGENE FOSTER, M.D.,
President of the Board of Health of Augusta, Ga.

The alarming and wide-spread prevalence of small-pox in the United States at the present time admonishes us of the necessity of ever keeping in view the measures proper to be applied to its prevention and control. In no disease in the whole catalogue of those celebrated for fatality has man such absolute mastery as of small-pox. Since the transcendent discovery of Jenner of the antidotal powers of vaccination there is not the shadow of an excuse for the continued existence of this horrid and extremely fatal disease. Had mankind been mindful of the duty of self-preservation, this malady would long since have been stamped out of existence. In 1698 Jenner gave to the world his glorious discovery, vaccinnation. For centuries prior to this time small-pox had been justly regarded as the king of fatal diseases. We are told by M. de LaCondomine that small-pox was the cause of one-tenth of all the deaths among mankind. He further says: "Among those who outlive it many either totally or partially lose their sight or hearing; many are left consumptive, weakly, sickly or maimed; many are disfigured for life by horrid scars, and become shocking objects to those who approach them. Immense numbers lose their eyesight by it." Half a million deaths were annually caused in Europe from small-pox prior to the discovery of vaccination. Macauley, in speaking of small-pox in England, says: "The havoc of the plague had been far more rapid, but the plague visited our shores only once or twice within living memory, but the small-pox was always present, filling the churchyards with corpses, leaving on those whose lives it spares the hideous traces of its power, turning the babe into a changeling at which the mother shuddered, and making the eyes and cheeks of the betrothed maiden objects of horror to her lover."

Rosen says that in Sweden one-tenth of all the deaths was from small-pox. We are assured that this disease concurred with fire and sword, and famine and blood-hounds, to complete the depopulation of St. Domingo.

In the sixteenth century small-pox fell upon Mexico, and in a few years three million five hundred thousand of the population yielded up their lives to it, leaving in some places scarcely enough people alive to bury the dead. Brazil, in 1653, was invaded by small-pox, and in some instances whole races of men were carried to their graves by it. The Province of Quito, in a few years, lost one hundred thousand of her Indian population by this one disease. In 1707 Iceland was invaded by small-pox, and desolation and ruin followed in its wake for years, causing in 1707 the death of eighteen thousand out of a total population of fifty thousand. The fearful fatality of the disease is evidenced by the following extract: "In one island they found one girl, with the small-pox on her, and her three little brothers; the father having first buried all the people in the place, had laid himself and his smallest sick child in the grave, raised with stones, and ordered the girl to cover him." Greenland, in 1734, lost more than two-thirds of her population by small-pox. We are told, also, that one-sixth part of the inhabitants of Ceylon died of small-pox. Siberia has nearly an equally blood-curdling history. Kamschatka has felt a like heavy hand from this terrible disease. One-tenth of all the deaths in France, prior to vaccination, were from small-pox. This country has been most cruelly and fearfully scourged by small-pox, the details of which must be too well known to need repetition here. Whole tribes of our Indian population were swept out of existence by it. Europe, in the century preceding the discovery of vaccination, lost in deaths from small-pox 50,000,000 of her population. But it is unnecessary to sicken the heart with recital of such details. Enough has been presented to justify Macauley in calling small-pox "the most terrible of all the ministers of death." Suffice it to say small-pox prevailed in all countries, spared neither the babe at the breast, the youth nor maiden, the father nor mother, the priest nor layman, nor the white heads of the old and trembling man or woman, for it was remorseless in the vigor with which it swept the aged into the grave. Whenever and wherever it appeared in the past, in a community unprotected by vaccination, it is well described by Alexander McKenzie, *i. e.*: "It was as a fire consuming the dry grass of the field. The infection spread with a rapidity which no flight could escape, and with a fatal effect which nothing could resist." This disease had but one feature which was not repulsive; that one was that it was no respecter of persons. Reaching to the loyal throne of France it laid the mighty Louis XIV. in the grave. In Mexico it treated the Emperor similarly. In England it invaded the household of William III., killed his wife Mary and several others of his family, and would not leave the palace until it had attacked the king and maimed and disfigured him for life. The hovel of poverty was in this respect the equal of the palace of wealth, for small pox was the terror of every household.

With all the fatality, loathsomeness, horrid disfigurement and permanent disabl ment consequent upon small-pox, need we wonder that the people fled their homes frenzied with fear; or that men reared in the school of the soldier, whose gallantry and intrepidity had been tested before the cannon of the enemy belching forth missiles of death, should cower before this the mightiest of engines of destruction known to man, and with faces blanched with fear, turn their backs upon it in precipitate flight. Longsuffering mankind eventually became hopeless of escape from the dread monster. When the brain of the civilized man was hopeless of escape from small-pox, need we wonder that the fiery and untamed savage despaired also? McKenzie says of the Indians: "It was not uncommon for the father of a family whom the infection had not reached, to call them around him, to represent the cruel sufferings and horrid fate of their relations, from the influence of some evil spirit who was preparing to extirpate their race, and to incite them to baffle death, with all its horrors, by their own poinards. At the same time if their hearts failed them in this necessary act, he was himself ready to perform the deed of mercy with his own hand, as the last act of affection, and instantly to follow them to the common place of rest and refuge from human evil."

After centuries of havoc from small-pox variolous inoculation was introduced and the mortality per thousand greatly lessened. But it was reserved for the philosphical mind of the immortal Jenner to evolve from the story of the humble milk-maids of a country district of England, the simple, harmless and glorious discovery of vaccination. Upon hearing the story of the milk-maids, their assertions of the protective powers of cow-pox against small-pox made an impression upon his mind which was the source of much thought. Believing in the possibilities of vaccination, he so persistently brought the subject to the attention of the medical society of which he was a member that he was threatened with expulsion as a bore. The year 1796 was the birthday of vaccination. With the scoffings and tauntings of the populace, who, in the recess of his great heart, he so yearned to benefit, called a villian for attempting to engraft the disease of the brute upon the human system; met with the assertion that small-pox was a necessary process of depuration to the blood, and that death should be visited upon him for his brazen impudence, Jenner dared to defend the right, and gave, through his trials and sorrows, to the world, without money and without price, the greatest blessing mankind has ever known. With all the violent opposition which he was daily meeting Jenner never flagged in his efforts to force this blessing upon his fellow man. Being invited to test his discovery he gladly accepted the invitation. After vaccinating a number of persons, and they going through the genuine vaccine disease, he subsequently inoculated them with the virus of small-pox, and failed to develop small-pox in a single one of them. To attempt to portray the fearful anxiety of this man as he was about to weigh his wonderful discovery in the stern scales of experience, or to picture the feelings of a heart filled with unspeakable joy in contemplating the benefaction which his genius was about to confer upon the human race, were an idle task. Within six years of this demonstration vaccination was known in nearly every portion of the world.

The sanitarian, looking to the prevention and control of small-pox, finds that his armory affords him the following weapons of defense: Inoculation, vaccination, isolation, disinfection.

*Inoculation.*—In no field of human research can a more curious phenomenon be found than is demonstrated in this measure. That you should have intentionally compelled a man to take small-pox in order to save his life is surprising indeed, but such was the intention, and the wonderful reduction of mortality among the individuals so treated fully testified to the propriety of the measure. Inoculation is the placing of the virus of small-pox, by puncture, under the skin, in the blood of the subject inoculated. The purpose of inoculation was to produce a modified form of small-pox which would afterward confer upon the subject so treated exemption from a malignant attack of small-pox. This weapon is of great antiquity. Kirkpatrick says of it, "Some poor, unlearned, but Heaven-taught mortal; some Chinese, Hindoo, or Circassain first hit upon it." It is admitted to have been practiced for long but unknown number of years in Hindoostan. The Chinese had for hundreds of years used crusts or scabs of small-pox, placed in the nose, for the purpose of inoculation. Of inoculation, in 1717 the wife of the English Ambassador to the court of Turkey, writing to her home says, "The small-pox, so fatal and general among us, is here entirely harmless by the intervention of engrafting, which is the term they give it. Every year thousands undergo the operation, and the French Ambassador says pleasantly, that they take the small-pox here by way of diversion, as they take the waters in other countries. There is no example of any one who has died of it." Dr. Simon says of this measure, "to the present time it remains one of the most interesting and least explained facts of pathology that the specific contagion or ferment of small-pox, so uncontrollable in its operations when it enters a man in the ordinary way of his breathing an infected atmosphere, becomes for the most part disarmed of its virulence when it is artificially introduced into the system through a puncture of the skin." By resort to inoculation the mortality among those so treated, was reduced from one death in every three cases of natural small-pox, to one death in every three hundred cases of inoculated small-pox. Some authorities give the protective powers of inoculation as representing one death in every one thousand cases. Thus it will be seen that the practice of inoculation offered to the individual doomed to take small-pox the greatest possible security against death of any measure in use prior to the introduction of vaccination. While the security against death to the individual in whom small-pox was inevitable was most wonderfully augmented by inoculation, yet to the country at large the practice of variolous inoculation was a curse instead of a blessing—for inoculated small-pox lost none of its fatal power of conveying malignant small-pox to all unprotected persons. The Royal College of Physicians, of London, in 1807 (report on vaccination) says, "However beneficial the inoculation of small-pox may have been to indivuals, it appears to have kept up a constant source of contagion, which has been the means of increasing the number of deaths by what is called the natural disease." Dr. Heberden says in the first thirty years of the 18th century before inoculation became general the deaths from small-pox to every 1,000 deaths were 74; inoculation for an equal period during the general practice at the end of the 18th century the deaths from small-pox were 95 to 1,000

deaths—an increase in the proportion of five to four. After the discovery of vaccination, it being so much superior to inoculation, the latter practice was made unlawful.

*Vaccination.*—Of this weapon of defense against small-pox, Simon, after speaking of the mysterious effects of inoculation, says of vaccination: "Equally strange and inexplicable is the further and greater change which this ferment undergoes in passing through the textures of a cow; a change which renders it incapable, when retransplanted to the human system, of any longer propagating itself by effluvia, while it retains its capability of propagation by inoculation, and its power of protecting the system against its own further action thereon."

Vaccination differs entirely in purpose and value from inoculation. The objects of vaccination are: 1. Prevention of small-pox; 2. Control of that disease when prevention is not obtained. As already seen, the only purpose or value of variolous inoculation was the mitigation of the severity of small-pox in the subject inoculated.

The first question which should be answered is, have we in vaccination a measure capable of effectually preventing the appearance of small-pox in man? Eighty-three years of experience upon the subject answers this question in the affirmative. Before attempting the demonstration of this proposition let us determine what proportion of mankind unprotected by vaccination is liable to contract small-pox if brought in contact with this poison. The experience of centuries unquestionably demonstrates the fact that the human race, with the exception of the merest fraction thereof, will in the course of a natural lifetime, contract small-pox if brought in contact with it. This proposition being admitted, let us appeal to the conclusive demonstrations of Jenner. After vaccinating six thousand persons he inoculated nearly all of them with the virus of small-pox, and failed to develop the latter in a single instance. What test could be so fair or thorough?

The difficulty of demonstrating this proposition from the records of civil populations is apparent to all, for vaccination of an entire population has never been known in a community of civilians. Dr. Makena describes an epidemic of small-pox in the Argentine Confederation from 1846-48—"as sweeping with the wings of death over that enormous tract of country, which extends from the seaboard of the Atlantic on the east, to the Corderilla of the Andes on the west. Throughout this whole space it may be said that hardly a single house or ranche escaped its fearful visitation, wherever the current of human intercourse reached, and such was its fatality, that I have known thirty children taken in one morning from the houses of one quadra of a street 150 yards long. Whole families were swept away, and, in short, the terrors of the plagues of former times were, if not surpassed, fully equalled by this horrible scourge. But that which struck me as most truly remarkable was, that not one of those English people who had been vaccinated at home, and who had the large, deep, oval thimble-mark on one or both arms, ever took the disease."

To go back to Jenner's day we find the following testimony from his papers: "From 1762 to 1792 the number of persons that died of small-pox in the Danish dominions amounted to 9,728. About the year 1802 vaccination was first introduced, and the practice became general, but not universal; however, fifty-eight persons died of small-pox in the year 1810. Vaccination, by order of the King, was now universally adopted, and small-pox inoculation prohibited, and from the year 1810 to the year 1819, not a single case of small-pox has occurred. From Bombay, I learn the small-pox is there completely subdued, not a single case having occurred for the last two years."

Drs. Seaton and Buchanan, in 1863, examined more than fifty thousand children in the national schools and workhouses in England to attempt to determine what proportion of them were unvaccinated, what proportion had formerly been vaccinated, and the number among those vaccinated who had contracted small-pox since vaccination.

Here is the tabulated results of that investigation:

| CLASSIFICATION OF CHILDREN. | No. Examined of Each Class. | No. in Each Class that had Traces of Small-pox. | Proportion Having Traces of Small-pox per 1,000 Children in Each Class Respectively |
|---|---|---|---|
| 1. Without any mark of vaccination | 2,837 | 1,010 | 360 |
| 2. With doubtful mark of vaccination | 508 | 30 | 59 |
| 3. With mark or marks of vaccination | 49,570 | 88 | 1.78 |

Dr. Marson, having the greatest experience perhaps of all writers upon this subject, says of the test of vaccination in preventing small-pox, " For thirty years we have revaccinated all the nurses and servants who had not had small-pox, on their coming to live at the small-pox hospital, and not one of them has contracted small-pox during their stay here."

Lord Jeffrey, in 1807, at that time editor of Edinburgh Review, a man most thoroughly skilled in the principles of searching and rigorous applications of the rules of evidence, highly endorses the value of vaccination, and says of Jenner's claim, " not until he had vaccinated some hundred children, and put them, at different intervals, to the test of inoculation for small-pox without effect, that he ventured to publish his discovery, in the year 1798, in a treatise, followed up the year after, by a still longer list of such experiments and observations." In the same article he adds, " When the practice of vaccination was discussed and confidently recommended, in 1800, by all the eminent practitioners in London, this was done only after full consideration of its efficacy, as compared to inoculation for small-pox; that Dr. Woodville, in particular, physician to the small-pox hospital, then stated that within the last six months he had vaccinated there 7,500 persons, the half of whom had been since inoculated with small-pox matter without the slightest effect being produced in any instance."

The report of the Faculty of Medicine at Prague to the Minister of the Interior, requesting information for the Government of Great Britain relative to the results of vaccination, offers the most interesting tables of any known to me. From this vast storehouse of facts the following summary is taken: From 1796 to 1802, inclusive, the average annual population observed was.............3,039,722
Total average number of deaths annually from all causes.................................. 94,955
Total deaths annually from small-pox........... 7,673
Showing one death from small-pox to every 396 of the population, and the deaths from small-pox to the total number of deaths was 1 in $12\frac{1}{2}$. From 1832 to 1855 inclusive, during 24 years subsequent to introduction of vaccination, with an average population of 4,248,155. Total deaths annually were on an average 131,412. The average number of deaths annually from small-pox, was 287,7-24; showing deaths from small-pox to population to be 1 in 14,741; deaths from small-pox to total number of deaths, 1 in 457. Among an average annual population of 143,122 persons vaccinated and 4,291 unvaccinated, the cases of small-pox annually existing were 389 among the vaccinated, and 355 among the unvaccinated population *i. e.:* One case of small-pox occurred among 367 vaccinated; one case of small-pox occurred among 12 unvaccinated. These tables further show the following facts: One fatal case of small-pox occurs among 7,166 vaccinated; one fatal case of small-pox occurs among 40 unvaccinated. In the Grand Duchy of Baden similar fruits have followed vaccination. Reliable statistics show that for a long number of years of compulsory vaccination with an average annual population of 1,200,000, only 100 cases of small pox occur each year, and only 13 of this vast population have died each year of small-pox.

In Great Britain from 1750 to 1800, of every 1,000 deaths, 96 were from small pox. From 1800 to 1850, of every 1,000 deaths, 35 were from small-pox. During the latter period the population was quite generally, but by no means universally, vaccinated.

In the German States where more attention is paid to vaccination the following were the results obtained: Before vaccination of population, deaths from small-pox amounted to 66.5 per 1,000; subsequent to vaccination 7.66 per 1,000.

Dr. Marson, of England, from the records of his great hospital experience, shows the merits of vaccination : "The small-pox death-risks of no-vaccination are to the death-risks of the very worst vaccination as three to one ; to the death-risks of the best vaccination as seventy to one."

In Norwich (England) in an epidemic of small-pox in 1819, Dr. Cross found that, in 31 families, those who had had cow-pox were living in the same room, or lying in the same bed with others suffering or dying from natural small-pox, yet remained perfectly safe, with the exception of only one child, whose mother reported that it had ten pustules."

From an experience of 21 years in Bohemia among four millions of population, the testimony of that country most strikingly illustrates the value of vaccination. Among the vaccinated population contracting small-pox the death-rate was but 5.1-10 per cent; the death-rate of the unvaccinated was 29.4-5 per cent.

The most reliable statistics, and at the same time the fairest, upon the value of vaccination, are to be found in the records of the army, for here alone is it possible to compel thorough vaccination of the entire population. Infringement of personal liberty, so dearly availed of as the shibboleth of the civilian, is treated with merited contempt in the army when it contravenes the rights of others. Vaccination having been decided an individual and collective benefit, the soldier has no choice in the matter ; he is compelled to submit to it. In thus protecting himself he at the same time benefits his comrades. To the honor of the surgeons of the army and navy be it said they are able, fearless and conscientious sanitarians, and in no other department have the rich results of sanitary science shown forth so conspicuously. Sir Gilbert Blaine says prior to vaccination, "Small-pox was one of the greatest embarrassments to the operations of armies." Let us see how it was after vaccination.

By reference to the statistics of sickness and mortality in the army of Great Britain for the twenty years from 1817 to 1836 inclusive, the following data are to be found : (Every soldier is vaccinated upon entering the army.)

In the dragoon regiments and guards, with an aggregate of 44,611 men, with a total mortality of 637, but three deaths occurred from small-pox.

At Gibraltar, with an aggregate of 60,000 troops, with a total mortality of 1,291, only one death was caused from small-pox.

Among the British and white troops in the West Indies, with an aggregate strength of 86,000, and a total mortality of 6,803; and among the black troops, numbering 40,000, with a mortality of 1,645, not one fatal case of small-pox occurred, although during this period several epidemics of small-pox decimated the islands.

Among the troops at Bermuda, Nova Scotia, Cape of Good Hope and Mauritus, for twenty years, there was not one death from small-pox.

In Western Africa, while small-pox was ravaging the inhabitants unvaccinated, not a case of small-pox occurred among the white soldiers who had been vaccinated.

From 1818 to 1836 inclusive, in an army of 40,000 aggregate, British troops at Malta, while small-pox was playing sad havoc among the unvaccinated inhabitants, in repeated epidemics, there were only two deaths from this disease in the vaccinated army.

During the same period in Ceylon, among the white soldiers, with a total mortality of 3,000, there were only four deaths, with eight cases, from small-pox, notwithstanding repeated epidemics of the disease among the natives.

In the British troops serving in the United Kingdom from 1859 to 1864 inclusive, the following were the results of vaccination:

| | |
|---|---|
| Total number troops | 473,483 |
| " " cases of small-pox | 664 |
| " " deaths from small-pox | 40 |

Showing the ratio per 10,000 of strength to be—

| | |
|---|---|
| Cases of small-pox | 14 |
| Deaths from small-pox | 0.84 |

In the British navy—home force—for the same period of time, 1859 to 1864 inclusive, the following data are furnished:

| Total Mean Strength | Cases of Small pox. | Deaths. | Ratio per 10,000 of Strength. | |
|---|---|---|---|---|
| | | | Cases. | Deaths. |
| 127,660 | 416 | 29 | 33 | 2.3 |

Since 1803 to 1863, among the thousands of vaccinated children admitted into the Royal Military Asylum of England, there has not been a case of fatal small-pox. This testimony is the more striking since the records show that during that time four deaths occurred among those who had previously had small-pox.

Similar data might be furnished to an almost unlimited extent, but the further elaboration of this point must be unnecessary.

Will one vaccination performed in childhood protect the subject from small-pox during his life? This is a most vital question, and the sanitarian should be prepared to answer it. Jenner believed that the person who had been successfully vaccinated was "never afterward assailable by small-pox." He afterwards said of vaccination as follows: "Vaccination, duly and efficiently performed, will protect the constitution from subsequent attacks of small-pox as much as that disease itself will. I never expected it would do more; and it will not, I believe, do less."

To the careful observer there can be but one opinion on this point, *i. e.*, that the hope and claim of the great discoverer of vaccination can never be realized. Far be it from my desire to pluck even one tiny flower from the garland of immortality which a grateful race has woven over the name of Jenner; rather would I join in peans of praise to him who, amid so much sorrow and persecution, gave to hopeless and suffering mankind the greatest blessing it has ever known. Years upon years of experience, however, demonstrates that Jenner's enthusiasm carried him too far. It is natural that the flush of victory which succeeded to the scorn and bitterness of a then ungrateful people caused him to hope too much of his discovery. Tested by the argument which he himself said "was best, engraven with the point of the lancet," it has long since been demonstrated that in a large proportion of those vaccinated, one vaccination will not protect the subject against small-pox during his life.

Let us call to our aid "that keenest of all arguments for or against the practice of vaccination—those which are engraved with the point of the lancet." To this purpose the following data are offered:

In Wurtemburg with a vaccinated population of 1,263, 298, during five years the total number of cases of small-pox was 1,667, (354 of these were cases of confluent small-pox, 1043 were cases of varioloid), being about one case of failure of protection against small-pox to 217 vaccinated persons. In a few years subsequently, of 44,000 revaccinated subjects 20,000 took the vaccine disease perfectly, 9,000 imperfectly and failed with 15,000. The successful revaccinations being almost exclusively in those who had been vaccinated many years previously.

Of 14,334 revaccinations in the army of Wurtemburg, 8,845 had what are described as genuine vaccine marks or scars, and of this number 31 per cent. were successfully revaccinated; aborted vaccine vesicles in 29 per cent.; and re-vaccination failed in 40 per cent. With those having imperfect marks of previous vaccination, revaccination succeeded in 28 per cent., modified in 26 per cent., failures 46 per cent. Mr. Simon, in his able digest of the subject, published by the General Board of Health, shows that, during the years from 1833 to 1837, that, notwithstanding the fact that small-pox had been sixteen times brought into the army of Wurtemburg, there had ensued among the 14,334 revaccinated soldiers one single instance of unmodified small-pox.

In the Prussian army in 1840, revaccination was performed upon 43,522 soldiers. Upon these soldiers were found distinct vaccine cicatrices in 34,573; indistinct vaccine scars in 6,177; in 2,772 persons no scars of previous vaccination were found although they had formerly been vaccinated. The result showed 20,952 successful revaccinations; 8,820 partially successful; and in 13,-750 unsuccessful revaccinations. Revaccination was practiced in this army because of the increase of the number of cases of post-vaccinal small-pox among the soldiers. For ten years prior to 1831 these cases had been observed, and from 1831 to 1833, 312 deaths had occurred among the troops formerly vaccinated. For twenty years subsequent to revaccination two deaths annually have occurred from small-pox, whereas 104 deaths annually occurred before revaccination was practiced.

In the Bavarian army revaccination has been compulsory since 1843, and for twelve years, 1843 to 1855, as shown by the report of the Minister of War, not a case of unmodified small-pox had occurred. A few cases of varioloid had occurred during this period of time, but not a single death from small-pox.

In the Russian soldiers at Kazan, the army being revaccinated, 18 per cent. of perfectly successful revaccinations were obtained.

From the army of Denmark during the years 1842–1847, 20,000 soldiers were revaccinated, nearly fifty per cent. of revaccinations being successful.

The armies of Sweden, of Baden, and the British army, offer incontrovertible testimony of the necessity of revaccination, and almost total exemption of the revaccinated from small-pox. It would be both interesting and instructive to give these details did the limits of this paper permit. But one more demonstration of the value of revaccination. From the Report of the Faculty of Medicine of Prague, the following facts are shown: From 1840 to 1855 inclusive, the average number of revaccinations annually, were 11,-617, 2-16. Average annual successful revaccinations, 4,506. Unsuccessful, 6,890. Unknown, 213. From this it appears that of every 100 revaccinations there were 38.3-5 successful, 59.2-5 unsuccessful and 1.4-5 unknown.

These data might be so multiplied as to cover hundreds of pages, but further proof of the necessity of re-vaccination seems altogether unnecessary. I have purposely refrained, as far as possible, from giving individual experiences, of myself or others. The object has been to take the testimony of men who have had wide fields of experience, from which deductions to be satisfactory, must come. The attitude of the profession at large is that of indifference, and in a goodly proportion absolute hostility, to revaccination. Physicians will examine the vaccine scar, no matter how long since vaccination, and gravely assure the patient, "You may nurse small-pox patients as much as you please without fear of taking the disease. That scar is undoubted evidence of your protection against small-pox." Such opinions are daily and hourly expressed in a community where revaccination is urged. In view of the data above given, the physician who can tell from the vaccine scar whether or not its possessor is protected against small-pox must be on a high plane of guessing to which few of his fellow practitioners can ever hope to attain. It requires but little investigation of the subject, and a very limited experience, either in re-vaccination or examination of patients suffering with small-pox, to convince the seeker after truth that the opinion was born of ignorance and nurtured in prejudice. There is not a physician living (and if Jenner were alive the same thing would apply to him) who can tell from the vaccine scar if or not the patient is protected against small-pox. The only way of knowing if or not the patient is fully protected by a former vaccination is the test of re-vaccination. The measure is simple, harmless, and, if duly performed, effectual. If the revaccination "takes" it is fortunate for the patient that the operation was performed; if unsuccessful no harm is done. Vaccination should be performed in early infancy, and invariably revaccination should be had when adolescence is reached. When a community is seriously threatened with an epidemic of small-pox every citizen should be re-vaccinated.

*Isolation.*—No matter how absolute the protection afforded by vaccination there will always be great and insurmountable difficulties in the way of enforcement of general resort to it in this country, where individual ignorance and prejudice are allowed to contravene the public good. Therefore the sanitarian must be prepared to enforce additional and different measures of control of small-pox. If a case, or cases, of small-pox be introduced into a community and isolation of patient, and disinfection of infected materials be forthwith required the disease cannot spread over the country.

Whenever a case of small-pox occurs in a family the first duty is to isolate the sick from the well, and provide nurses and attendants who have been carefully vaccinated. If others in the family have not been previously vaccinated separate them from the sick and vaccinate them at once. Those who have been previously vaccinated should be promptly revaccinated. If the small-pox patient be where he can be isolated and provided with proper attention he may be permitted to remain at home for treatment, but the sanitary authority must furnish a guard to compel isolation. If isolation at home is impossible then a properly organized hospital is the only place where such patient can, with due regard to public safety, be treated. Where it is possible to do so, the name of every person exposed to the contagion by contact with the patient or infected material, should be promptly ascertained, and every such person vaccinated, and those already vaccinated should be revaccinated. These persons should be kept under observation for three weeks and if taken sick promptly isolated. Where small-pox prevails in a district, rigid house to-house examinations should be made in order to search out infected persons and compel prompt compliance with all necessary sanitary precautions. The sanitary authority must compel prompt reports of cases of small-pox if it would stamp out the disease immediately. This information should be required of the physician in attendance, and of the householder. The only way to obtain these reports is to prosecute to the utmost penalty of the law all delinquents.

It should be regarded as an especial and solemn duty upon the part of the authorities to annually provide gratuitous vaccination for the indigent poor, and whenever small-pox threatens the community no expenditure of money, within the line of duty compatible with the public good, should be spared by the authorities in order to protect the people under their charge. To this purpose a suitable hospital is indispensable. It should be located in a place remote from the thickly settled part of the city. This institution should be provided with every necessary comfort, and its unfortunate and dangerous inmates should be so considerately and tenderly cared for that the people will learn that it is a haven of refuge into which the afflicted may gladly enter and find shelter and protection—not a prison, where the wants and needs of these loathsome victims are cruelly and wantonly ignored. In every house where small-pox is allowed to remain a large poster should be placed on the door to warn people not to enter or linger there.

*Disinfection*—Dr. Watson truly says, "there is no contagion so strong and sure as that of small-pox." The contagion of small pox is thrown off from the mucous and cutaneous surfaces of the person under the disease. Therefore, the contagious matter is found in the exhalations, the secretions and excretions and in the contents of the vesicles, pustules and scabs of the small-pox subject. These sources of disease contaminate the air of the sick room, and strongly adhere to everything therein. The vitality of this poison is truly remarkable if protected from the air, having in some instances, where infected clothing had been closely packed away, retained its power of infection for several years.

The sanitarian has in chemistry, under these surroundings, an indespensable ally. There is no question of our ability to kill every particle of the contagion of small-pox if chemical action is properly adjusted to its task. To do this successfully disinfectants must be brought directly in contact with the contagion of small-pox. Every discharge which passes from the mouth, nose, kidneys or bowels of the patient must be received in suitable receptacles charged with a powerful disinfectant Every handkerchief, towel, sheet, article of clothing, drinking or eating vessel, used by the patient must be purified by heat and disinfectants. If the patient is kept at home a sheet saturated with a strong solution of carbolic acid (4 ozs. to quart of water) should be hung over the door openings outside the sick room, to prevent the contagious exhalations from passing into other parts of the house.

The sputa, excretions of the kidneys and bowels should be received into vessels containing a sufficient quality of strong copperas water, 2 lbs to the gallon of water. Under no circumstances should these excreta be passed into a water closet or thrown into the privy vault or yard until an additional quantity of copperas water be added to them. The quantity of the disinfectant should be at least eight times more than the excreta.

All eating or drinking vessels used in the sick room should be thoroughly disinfected with one of the above disinfectant solutions and afterward washed in hot water before carried from the room. Such of the articles as are necessary should be kept and used in the sick room until the recovery and discharge of the patient.

Every piece of bed or body linen from the patient or nurses, or any part of the sick room, must be kept in a carbolic acid and water solution for two hours, and boiled in water for half hour, before leaving the patient's room.

Use bits of cloth instead of handerchiefs, and burn them immediately when soiled.

The scabs and scales falling from the patient are powerfully contagious, the body of the patient should be daily coated with a solution of glycerine, or oil, with a small quantity of carbolic acid, (liquid carbolic acid ℥ ss, glycerine or oil ℥ xvi ) These oleaginous applications should be daily washed off with warm water and soap.

The patient should not be regarded as free from power of infecting others until every scab or scale has been removed. The hair should then be carefully combed so as to remove all particles adhering to the scalp. The whole body should then be washed in a weak disinfectant solution and the patient should hastily go into an adjoining room and dress with clothing undoubtedly free from contagious particles.

Immediately upon patient leaving the room it should be disinfected and cleaned. To effectually purify the clothing, bedding, etc., hang every article therein upon lines hung across the room. If there is a carpet on the floor take it up and burn it in the fireplace. Then stop up the fireplace and shut down the windows. Put two pounds of sulphur in an iron pan over a larger vessel holding water, and drop a quantity of live fire coals into the dish containing the sulphur, and then hastily leave the room, and close the door behind you. After being thus disinfected the room should be closed for twenty-four hours, in order that the disinfectant thus generated may invade every crevice and fully impregnate all materials therein. After twenty-four hours open the windows (closing all others adjacent thereto), and then have the clothing and bedding which can be washed put in tubs containing solutions of carbolic acid, zinc or copperas, and then boil for one hour in water. These articles should then be taken into a large open space, as far from the thickly inhabited portion of the town as possible, and sunned. There is always danger in the pillows and mattresses, for they cannot be thoroughly fumigated nor can they be washed, therefore these articles had better be burned. All stuffed furniture in the room should be torn off and such stuffing materials burned. Wash all furniture and the floor in a disinfectant solution. If the walls be papered wash off every particle of such covering and then whitewash the walls. The walls, even when painted, should be washed with full disinfectant solution. After the above precautions are taken, open the windows for free ventilation and leave the room unoccupied for one month.

If the patient should die, the corpse should be thoroughly washed with a strong solution of zinc or carbolic acid and water, and the body should be wrapped in a sheet fully saturated with one of these disinfectants, and buried at once. Under no circumstances should the body of such a patient be permitted burial by public funeral; nor should the corpse be transported from the city by public conveyance—such as cars, boats, etc. Every hearse, hack, or other vehicle, in which the body has been conveyed, should be disinfected under the supervision of the sanitary authority immediately after the corpse is removed therefrom.

In the management of pauper cases the greatest possible care is necessary upon the part of the authorities. When a case has been found and is to be conveyed to hospital, this should be done in a covered ambulance. Infected clothing and bedding may be taken with patient to hospital, though prudence dictates burning them. The same measures as to disinfection of rooms and furniture and whitewashing walls must be rigidly carried out under the immediate supervision of the sanitary authorities. When infected persons or materials are being taken to hospital in a close ambulance (and it is criminal negligence to use an open conveyance), the ambulance should be driven through the most sparsely settled portions of the city contiguous to line of hospital, and the police should go ahead and clear the streets until ambulance has passed. Wherever the clothing and bedding of the poor have been burned, the public treasury should replace them, for they were destroyed for the public good. In burning these infected articles and replacing them with new ones, the corporate authorities have made a most prudent and economical expenditure of money—an expenditure which will return to them more than a thousandfold.

If small pox is to be stamped out of a community, the first cases must be promptly dealt with. Learn the lesson of the firemen in the control of the fire which would destroy your home, and in controlling this disease realize with them the terse language of Shakspere,

> "A little fire is quickly trodden out,
> Which, being suffered, rivers cannot quench."

In waging warfare against small-pox no alarm or excitement must be felt; meet it calmly and deliberately, and if real skill shall dir ct, and scrupulous conscientiousness shall execute, the above specified measures of prevention and control, you might simultaneously introduce small-pox into every city in America, and it would be impossible for it to attack any one citizen not already infected.

